# Automatic Assessment of Aphasic Speech Sensed by Audio Sensors for Classification into Aphasia Severity Levels to Recommend Speech Therapies

**DOI:** 10.3390/s22186966

**Published:** 2022-09-14

**Authors:** Herath Mudiyanselage Dhammike Piyumal Madhurajith Herath, Weraniyagoda Arachchilage Sahanaka Anuththara Weraniyagoda, Rajapakshage Thilina Madhushan Rajapaksha, Patikiri Arachchige Don Shehan Nilmantha Wijesekara, Kalupahana Liyanage Kushan Sudheera, Peter Han Joo Chong

**Affiliations:** 1 Department of Electrical and Information Engineering, Faculty of Engineering, University of Ruhuna, Galle 80000, Sri Lanka; 2 Department of Electrical and Electronic Engineering, Auckland University of Technology, Auckland 1010, New Zealand

**Keywords:** aphasia, severity level, deep neural networks, mel frequency cepstral coefficients, audio sensors, speech therapy

## Abstract

Aphasia is a type of speech disorder that can cause speech defects in a person. Identifying the severity level of the aphasia patient is critical for the rehabilitation process. In this research, we identify ten aphasia severity levels motivated by specific speech therapies based on the presence or absence of identified characteristics in aphasic speech in order to give more specific treatment to the patient. In the aphasia severity level classification process, we experiment on different speech feature extraction techniques, lengths of input audio samples, and machine learning classifiers toward classification performance. Aphasic speech is required to be sensed by an audio sensor and then recorded and divided into audio frames and passed through an audio feature extractor before feeding into the machine learning classifier. According to the results, the mel frequency cepstral coefficient (MFCC) is the most suitable audio feature extraction method for the aphasic speech level classification process, as it outperformed the classification performance of all mel-spectrogram, chroma, and zero crossing rates by a large margin. Furthermore, the classification performance is higher when 20 s audio samples are used compared with 10 s chunks, even though the performance gap is narrow. Finally, the deep neural network approach resulted in the best classification performance, which was slightly better than both K-nearest neighbor (KNN) and random forest classifiers, and it was significantly better than decision tree algorithms. Therefore, the study shows that aphasia level classification can be completed with accuracy, precision, recall, and F1-score values of 0.99 using MFCC for 20 s audio samples using the deep neural network approach in order to recommend corresponding speech therapy for the identified level. A web application was developed for English-speaking aphasia patients to self-diagnose the severity level and engage in speech therapies.

## 1. Introduction

### 1.1. Background

Stroke is a condition that occurs with the death of brain cells due to poor blood flow toward certain parts of the brain [1]. There are mainly two categories of stroke: ischemic occurring due to the lack of blood flow, and hemorrhagic occurring due to bleeding [2]. If the affected area of the brain after a stroke involves frontal lobe or the parietal lobe, it can produce symptoms related to aphasia [3]. This paper targets patients diagnosed with speech disorders due to aphasia followed by a stroke. Additionally, aphasia can be caused more slowly by brain tumors, head injuries, brain surgery side effects, and brain infection or neurological disorders such as dementia [2].

A person with aphasia can have trouble speaking, writing, reading, and also understanding the language. These implications can range from mild or simple to very severe or nearly impossible to communicate. Some aphasia patients only have trouble in one area of communication such as collecting words together to form meaningful sentences, troubles in reading or understanding what others are saying [4]. Nearly all people with aphasia have word-finding difficulties such as coming up with the correct name of people, places, things, or events.

There are both general and specific treatment options for aphasia. The treatment procedures can specifically vary with the language profile of each individual. Community support includes aphasia groups which are groups that offer the individuals and the family members the opportunity to socialize [5]. Life participation support to aphasia helps aphasia patients to reengage and rearrange their lives through daily participation in activities [6] with consistent motivation, and a dependable support system that requires full participation [7]. Computer-based treatment involves specially created mobile applications and touchscreen devices that target various language skills [8]. Constraint-Induced Language Therapy (CILT) is a procedure to discourage the use of compensatory communication strategies and encourage the use of spoken language [9]. In the Melodic Intonation Therapy (MIT) approach, the musical elements in the speech such as melody, rhythm, and stress are used to improve the expressiveness of the language. This is used often for individuals with severe and non-fluent aphasia [10]. Augmentative and Alternative Communication (AAC) is a method of treatment that can supplement or replace natural communication with aids such as pictures, symbols, objects and/or unaided symbols such as finger spelling, gestures, and manual signs [11]. Visual Action Therapy (VAT), which is used often with patients with global aphasia, is a nonverbal treatment that trains patients to use hand gestures to indicate the visually absent items having a 12-step training hierarchy [5]. Conversational coaching is designed to train the verbal and nonverbal communication strategies to the patients. These strategies can include drawings, gestures, cues, confirming information, and summarizing information [5].

Digital solutions exist which can be used to treat aphasia. Camelendar is a touchscreen application that supports people with aphasia in conversation [12]. Vithea is an online system that uses Automatic Speech Recognition (ASR) to verify words spoken by the patient where patients can practice repetitive exercise sessions with simple instructions while staying in their comfort zone [13]. EVA Park is an online virtual world that gives aphasic patients an equal opportunity to practice sessions and make social connections [14]. Self-management applications consist of pre-recorded practice sessions, where the therapists can monitor the performance of patients against standardized performance metrics [15]. Everyday Life Activities (ELA) is a virtual house designed with the objective of integration of face-to-face language therapy and computer graphics-based virtual applications to administer aphasia patients [16]. Web-ORLA is a tele-rehabilitation application that uses information and communication technology for rehabilitation purposes, where the 3D animated character is used to talk with patients with the help of recorded messages [17]. Some have attempted to improve the speech of aphasia patients by creating a visual storytelling application that consists of photos, drawings, icons, etc. which can be saved and shared [18].

Sensors have been extensively used in applications related to aphasia. As aphasic patients have difficulty expressing their needs, researchers have attempted to assist them using gesture detection with the help of machine learning, using the input received from wearable sensors [19]. Some have even attempted to detect sign language using Wi-fi channel state information which does not require the user to wear any sensor [20]. Electroencephalography (EEG) signals recorded synchronously with aphasic speech have been used to improve the performance of a deep learning based automatic speech recognition system [21]. EEG signals have also been used to assess the sleep slow wave activity of aphasia patients after an imitation-based speech therapy [22]. Some have used Radio Detection and Ranging (RADAR) equipment in a bedroom scenario consisting of RADAR transmitters and sensors to detect the hand gestures of aphasia patients using transfer learning [23]. Cameras have been used as sensors to capture images of aphasia patients to aid them in speaking with the help of speech therapists, as shown in research conducted in [24]. A magneto-inductive sensor array placed outside the mouth and a permanent magnet placed on the tongue have been used to track tongue movements of aphasia patients during their speech-language therapy [25].

Natural Language Processing (NLP) is processing natural language such as spoken voice or text in order for computers to understand language in the manner humans can understand [26]. Automatic Speech Recognition (ASR) is one of the main use cases of NLP which recognizes speech by converting analog voice sensed by microphone sensor into a text or equivalent format [27]. For obtaining accurate outputs for NLP or ASR systems, the quality of the electrical output of the acoustic sensor should be high [28]. Early ASR systems have used a hidden Markov model with a Gaussian mixture model [29]. However, most of the modern ASR systems are based on deep learning models [30]. In most of our devices, we use some part of ASR. Google Assistant, Amazon Alexa, Apple Siri, and Cortana from Microsoft are some examples in which ASR is employed [31]. In the research literature, we can find many pieces of research that have been carried out concerning this regard, which implies the broadness of the research area. Now, there are ASR systems that can understand what language the speaker speaks within an instant, which is such an important achievement in ASR [32]. Hinton et al. in [33] describe in depth the past and present methodologies used in ASR. In order to understand and distinguish speech, there are several features that should be extracted and fed into the neural network for deep learning models such as Linear Prediction Coefficients (LPC), Linear Prediction Cepstral Coefficients (LPCC), Line Spectral Frequencies (LSF), Discrete Wavelet Transform (DWT), Mel Frequency Cepstral Coefficients (MFCC), and Perceptual Linear Prediction (PLP) [34]. However, one of the key features that the neural network should extract is the MFCC [33], as MFCC computation is a replication of the human hearing system intending to artificially implement the ear’s working principle with the assumption that the human ear is a reliable speaker recognizer [35]. Furthermore, researchers in [36], ref. [37] have used MFCC for aphasic feature extraction. Hence, for aphasic speech categorization, we can use MFCC to obtain better results.

ASR systems developed to recognize normal speech cannot be applied to detect impaired speech [38]. Amyotrophic Lateral Sclerosis (ALS) also known as Motor Neuron Disease (MND) is a disease that is caused by the progressive degeneration of motor neurons [39]. It has been challenging to recognize impaired speech due to defects in the articulation, breathing, voicing, and prosody of the patients with disordered speech [40]. Using robust phonetic elements with the individual speaker output patterns of ALS patients, the STARDUST project is an ASR system developed for people with severe dysathria using hidden Markov models [41]. Some have attempted to fine-tune the size and shift parameters of the spectral analysis window of the short time Fourier transform of an ASR system to reduce the error rate of dysarthric speech [42]. Some have used ASR for assistive writing in ALS patients as a supplementary tool for word prediction when the first letter of a word is typed, and the rest of the word is predicted using the ASR [43]. Project Euphonia by Google is another attempt to identify impaired speech to be better understood by others [44]. Parkinson’s disease (PD) is a progressive brain disorder having speech defects such as slurring words, mumbling, trailing off at the end of a sentence, etc. [45]. A deep dual side learning ensemble model, combining a deep sample learning algorithm and an embedded deep stack group autoencoder, has been used to recognize the speech of patients with Parkinson’s disease [46]. A cross-language classification of people as Parkinson’s disease patients and healthy speakers has been completed in [47] using ASR. Another similar study uses acoustic features, prosodic features, and features derived from syllable repetitions, text reading, etc. to detect Parkinson’s disease with the help of Support Vector Machines (SVMs) [48]. Sakar et al. in [49] have used machine learning to diagnose Parkinson’s disease by using a voice dataset collected from such patients. In a tablet-based therapy for aphasia, ASR has been used to provide feedback to the patient to improve the speech of aphasia patients [50]. A system to improve the quality of speech in aphasia patients using “processing prosthesis”, which is a software that allows users to record speech fragments and build them into larger structures by manipulating visual icons, has been used in combination with an ASR system [51].

Speech error detection is a research area that detects speech errors either at the word or phonetic level [52]. One of the similarities between ASR and speech error detection is the correct identification of audio feature sets to feed into the system to obtain accurate results. The authors of [37] have assessed the speech error rates of Cantonese aphasic patients in which the authors have used two methods to obtain their assessment. The first approach uses the Gaussian Mixture Model–Hidden Markov Model (GMM-HMM), and the second method uses the deep neural network–Hidden Markov Model (DNN-HMM) to test the syllable error rates on the CanPEV dataset and the Cantonese Aphasia-Bank dataset [53]. According to the results in [37], the performance of the DNN-HMM is better than that of GMM-HMM, and the performance advantage of the deep neural network-based architecture also diminishes the time that it takes to process the audio sample. Another similar study assesses the syllable error rates of aphasia speakers and unimpaired speech, attempting to quantify the impairments [54]. In such works, the authors also suggest that the spontaneous nature of speech affects the model performance, and the feature sets that were used to extract the acoustical features are not analyzed in their work. The study in [55] also has a similar scope as the previously discussed studies [37,54], but it uses a mixture of intelligent experts to find the error rates of speech. A lower syllable error rate for aphasic speech has been obtained by using a Time Delay Neural Network (TDNN) along with a Bidirectional Long Short Term Memory (BLSTM) for acoustic modeling in ASR [56].

### 1.2. Motivation

A semi-supervised learning approach to recognize the speech of English and Spanish aphasia patients has been conducted in [57]. ASR has been used to diagnose patients suffering from primary progressive aphasia by extracting features from transcripts combined with acoustic features of the speech signals [58]. In research conducted in [36], the AphasiaBank data set has been used in an ASR system where they have observed an increasing syllable error rate with the increment of the severity of aphasia. However, the researchers in [36] still have not been able to come to a conclusion to classify the research into aphasia levels based on syllable error rates. Very recently, Marjory et al. have used machine learning to classify aphasic speech into four categories (mild, moderate, severe, and more severe) [59]. Another similar study uses the NLP of transcripts to predict three severity levels of primary progressive aphasia [60]. However, the speech therapy recommendations from such systems can be argued to be more generic, and the given four categories may not map to four categories of aphasia (global, Brocas, Wernickes, anomic). Some researchers have used Artificial Neural Network (ANN) to diagnose Alzheimer disease, which is characterized by communication deficits including aphasia [61]. Automatic speech assessment is another research area that focuses on the efficient assessment of the aphasic speech. A recent research study in [62] suggests a method of automatic speech lucidity assessment for Mandarin-speaking aphasic patients using machine learning-based techniques, where quadrature-based high-resolution time frequency images with a Convolutional Neural Network (CNN) are utilized to develop a method that can map the relationship between the severity level of aphasic patients’ speech and the three speech lucidity features.

In this paper, we propose a method to classify the severity level of the aphasic speech of the aphasia patients with the aid of machine learning and speech feature extraction. In severe stages of aphasia (global aphasia), oral motor exercises and simple sound exercises are used as speech therapies [63,64,65]. At later stages, exercises are focused on pronouncing words, building vocabulary, and continuing a conversation [66,67,68,69,70,71]. Vithea [13] application is capable of training patients with severe Broca’s aphasia. Furthermore, none of these applications reviewed in the literature are specially designed for patients with global aphasia. All these applications are designed using conventional software design principles. They do not have the power of data-driven decision making to identify the Aphasia severity level. Aphasic speech processing is the only research field that uses the power of machine learning technologies instead of conventional approaches. The correct classification of an aphasic patient is very important for the patient’s speech therapy process. That is because it allows the patient to be treated with the therapy exercises that are appropriate for the patient’s aphasia severity level [72]. Otherwise, a patient may be given a therapy exercise that is too hard or too easy. In none of these cases can we expect any improvement in the speaking of the patient. In practice, a trained medical practitioner will assess the speech of the patient by how well or worse the patient performs in a given set of exercises [73]. This process may take up some time, and the patient would have to participate in many sessions with the practitioner before the correct category is identified. Automating the aphasia detection process allows practitioners to directly start the speech therapy of the speech, thus reducing the time of recovery. Such automated systems should be sensitive to subtleties of defective speech, and the extraction of an effective set of features is very important [74].

In this research, we automate the task of distinguishing different levels of aphasic speech, which is very much important in cases such as speech therapy, where the patient’s level has to be determined before assigning treatment [75]. None of the existing methodologies provide a comprehensive approach for assessing the speech defect levels of the aphasia. However, we propose ten severity levels, which are based on absence or presence of a set of speech features, which map to ten different types speech therapies found in the literature. In contrast to our methodology, most of the authors of the reviewed literature have tried to assess the syllable error rate of speech for aphasia patients [36,37,54,56]. However, speech assessment is much more important with regard to the patient, because correct assessment allows the therapist to perform speech-language therapy on the patient within a short amount of time [76]. With the correct set of audio features, the prospect of using machine learning to automate aphasia detection and classification is tested in our methodology. To the best of our knowledge, we are the first to classify aphasic speech to ten severity levels for speech therapy recommendation using a machine learning and speech feature extraction-based approach.

### 1.3. Problem Statement

The research problem is the lack of an automated tool to classify aphasia patients based on speech fluency in order to recommend speech therapies based on the fluency.

### 1.4. Objectives

- To identify a set of audio features and to identify the ideal length of an audio frame for aphasia detection;
- To categorize patients into aphasia severity levels based on speech fluency (presence or absence of speech features);
- To map identified aphasia severity levels into aphasia categories and corresponding therapies;
- To evaluate the performance of different machine learning models for aphasia level and category detection;
- To develop a software application to automatically recommend speech therapies to aphasia patients using the machine learning model and speech feature extractor having the best classification performance.

### 1.5. Benefits to Community, Healthcare and Social Value

The research findings will play a vital role in aphasia level classification. The speech improvement of aphasia patients is evaluated by speech and language pathologists manually. Since there is only a limited number of speech and language therapists, patients receive this evaluation only during their therapy sessions. In our research, this speech assessment process is automated using machine learning and acoustic feature extraction. With this automation, patients can do more personalized training sessions with correct evaluation metrics even at their homes. This will cause a greater reduction in health care costs for patients and states. In the healthcare sector, speech and language pathologists are responsible for patient rehabilitation activities. Due to the growth of patients each year, there is a huge demand for assistive tools for health care professionals. With the automation of aphasia level identification, pathologists can reduce the monitoring time of the individual patient and can access more patients within a day. Furthermore, due to the automatic detection of severity levels, patients can be given personalized home training sessions, which causes a greater improvement in the process of rehabilitation of aphasic speech defects.

### 1.6. Contribution to Existing Literature

In the existing literature, there are solutions for aphasic speech recognition and assistive tools for speech training. The missing piece is that none of these methodologies focus on automating the identification of aphasia severity levels based on speech fluency having distinct speech characteristics targeted for speech therapies. Identification of the correct level of the aphasia patient is very important, as it enables giving more specific training for that level, which causes the rapid progress of the recovery process. According to our best knowledge, we are the first to identify ten aphasia levels based on the speech fluency of aphasia patients as well as speech therapies for each level, and we are also the first to experiment on the use of different machine learning and speech feature extraction techniques for aphasia level detection in order to recommend speech therapies specific for the identified aphasia levels. Furthermore, we are the first to experiment on the effect of the length of the input aphasic audio for aphasia level classification and category classification performance. In this research, we exploit the use of machine learning for the classification of aphasia level and aphasia categories. This opens doors for new research areas such as aphasia assistive tools that have the capability to identify the defective level of the patient and recommend speech exercises accordingly.

## 2. Materials and Methods

### 2.1. Ethical Approval and Adherence to Guidelines and Regulations

Ethical approval was obtained in writing from the Aphasia Bank [77] owners to obtain access to the database. There are ground rules (basic rules for data sharing, principles of data sharing, code of Ethics of TalkBank for maintaining confidentiality, etc.) for using the Aphasia Bank database [78]. All authors have followed the ground rules in using the database for this research. Speech recordings obtained from the database were confidentially used for the research. As the research did not involve obtaining samples of data using live aphasia patients for training or testing of the proposed model, ethical approval from an ethics review committee is not required. All experiments were conducted using the data obtained from Aphasia Bank without the involvement of live patients. All experiments were performed in accordance with relevant guidelines and regulations (Declaration of Helsinki, SAGER guidelines). Informed consent has been obtained from all the participants in the Aphasia Bank database before their recordings have been obtained. There are no legal/social/financial issues for this research.

### 2.2. Identifying Severity Levels of Aphasia and Mapping to Aphasia Categories

Usually, the aphasia patients are classified into four/five severity levels. The Aachen Aphasia Test (AAT) [79], which is the gold standard for diagnosing and grading aphasia syndromes, also classifies an aphasia patient into five classes based on severity. However, several researchers have shown that traditional standardized aphasia tests may not be sensitive enough to adequately assess linguistic deficits and recovery patterns in persons with aphasia to recommend speech therapies [80,81]. That is because different sub-levels can be identified within the aphasia categories of the standard tests such as AAT, which is based on linguistic deficits. For example, the severity of the Broca’s aphasia can be subdivided into two levels: severe Broca’s aphasia and mild Broca’s aphasia [82]. Therefore, two sets of speech therapies can be identified for Broca’s aphasia patients based on the severity. If the machine learning model can only identify Broca’s aphasia from Wernicke’s aphasia, both mild and severe Broca’s aphasia patients will be recommended the same speech therapy by the automated tool. However, in our methodology, we distinctly identify severe Broca’s aphasia (using level 4) and mild Broca’s aphasia (using level 5) such that specific speech therapy for identified aphasia severity level can be recommended. The novelty of this research exists for the aphasia severity level identification and mapping the identified severity levels into aphasia categories and speech therapies. We define aphasia severity level as an aphasia stage which can be distinctly identified using the absence or presence of speech features. We identify the following features of speech targeted for speech therapies in order to identify the severity levels of aphasia. They are,

- SF—Swallowing Function;
- LM—Lip Movement;
- TM—Tongue Movement;
- AS—Any Sound;
- RSTS—Repeat Single Tone Sound;
- RVS—Repeat Vowel Sounds;
- RWOCS—Repeat Word or Compound Sound;
- PCWOS—Pronounce Comprehensive Word or Sound;
- RGSWE—Repeat Given Sentence with Errors;
- RSSCWE—Repetitively Sing Song Chorus with Errors;
- RSWAQ—Respond Single Word Answer Questions;
- RCWE—Respond Constructively with Errors;
- RCAF—Respond Constructively and Fluently.

Using the absence or presence of the above-mentioned speech characteristics, aphasia patients are classified into severity levels as shown in Table 1.

What is found in the clinical literature are the four aphasia categories, which are namely global aphasia, Broca’s aphasia, Wernicke’s aphasia, and anomic aphasia. We can map the aphasia main categories with the identified aphasia severity levels as shown in Table 2. Global aphasia is characterized by inability or extreme difficulty in speaking, reading, and writing [83]. Therefore, levels 0 to 3 include those who can at most produce single vowel sounds, and these levels map to global aphasia (Category 1). Broca’s aphasia caused by damage to Broca’s area in the brain is characterized by the ability to speak words but inability to complete sentences and not being able to express in the manner desired [84]. Hence, levels 4 and 5 include those who can at most speak words, and these levels map to the Broca’s aphasia (Category 2). Wernicke’s aphasia caused by damage to Wernicke’s area in the brain is characterized by the ability to speak in long sentences, which usually do not make sense, as they do not understand words. The spoken words in the sentences are characterized by wrong words or nonsense words [85]. Therefore, levels 6 and 7 include those who can at most repeat the chorus of a song with errors, and these map to Wernicke’s aphasia (Category 3). Anomic aphasia patients have relatively preserved speech fluency, repetition, comprehension, and grammatical speech, but they have difficulty with word finding [86]. Therefore, levels 8 and 9 include those patients who can at most respond to a question constructively with errors, and these levels map to anomic aphasia (Category 4).

As evident from Table 2, an aphasia category is an abstraction of the aphasia severity levels. In other terms, several aphasia severity levels are combined to form an aphasia category. One can recommend speech therapies based on the aphasia category also to a certain extent. However, as evident from the severity levels, aphasia categories can be subdivided to form speech levels based on the absence or presence of speech features. Therefore, to recommend an accurate therapy, the identification of levels is required.

### 2.3. Mapping Aphasia Severity Levels to Speech Therapies

We map the identified aphasia levels with speech therapies found in the literature as shown in Table 3.

### 2.4. Data Collection

An online database for aphasia patients from Aphasia Bank [87] was used for the data collection process. Aphasia Bank is a very large database that contains video and audio recordings of aphasic patients along with transcripts of the conversation. After thorough examination of the database, a sizeable collection of data (video recordings) was collected for training purposes. These video recordings included therapy sessions conducted by a speech therapist with an aphasic patient that involved several speaking exercises. The therapy sessions contained exercises such as asking questions from the patient where the patient needs to respond, picture description tasks, story telling and sentence repeating, etc. The recordings have been taken while the patients were having a language assessment test with a trained speech therapist. Prior to recording, patient consent has been taken to participate in the speech therapy. We can find aphasic patients from many categories in the database, but the most prominent group belongs to category 2 aphasia. The database does not contain data for severe levels of global aphasia such as level 0 and level 1. Male representation in the database is high, as many recordings had male patients. In addition, the database had very few representations of persons with color where most of the patients were Caucasian. The database also contains recordings for other language patients beside English-speaking patients.

### 2.5. Data Sample

The sample consists of a set of recordings selectively obtained from the Aphasia Bank database subjected to inclusion and exclusion criteria.

Confidentiality and anonymity—The patient recordings in the Aphasia Bank database do not contain any identifying information of the patients. The patient recordings have been named using the therapist’s name.

Participants—Participants are aphasic patients belonging to different aphasia severity levels.

Inclusion and exclusion criteria—All English-speaking recordings were included. All other language responses were excluded.

Sample size—The sample consisted of 54 patient recordings broken down into 1555 20 s chunks and 2940 10 s chunks for training and testing purposes.

### 2.6. The Proposed Aphasia Classification Model

**Figure 1:**
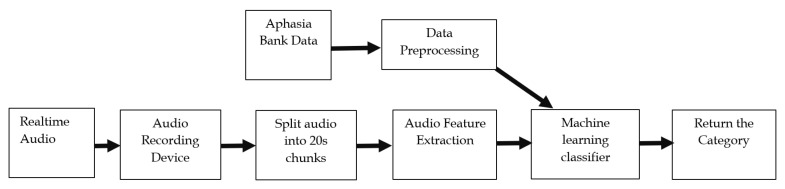
Aphasia classification model block diagram.

The main steps of the proposed aphasia classification model are shown in Figure 1.

As evident from the block diagram given in Figure 1, we implement the classification model for two scenarios. The first one is for initial training and testing purposes, which uses the collected data set from Aphasia Bank that is preprocessed as specified in the following section and input to the machine learning classifier. The second scenario is a real-time voice input, which is recorded by a recording device and fed into the machine learning classifier. Even though we have implemented the system to obtain real-time inputs, we have completed all our research experiments using the first scenario, which is by using the data set collected from Aphasia Bank. Unfortunately, we could not find any aphasia patient in Sri Lanka who speaks the English language to be tested using the real-time system. However, we have included the real-time system in our software application to be used by any interested researcher/patient worldwide to make use of it.

#### 2.6.1. Data Preprocessing

The data set has been subjected to several preprocessing steps and then used to develop the model. The data set that was collected from the Aphasia Bank had to be subjected to the following preprocessing steps, as it was a video data collection that was intended to be used for medical purposes. Therefore, the data set had to be prepared in such a way that it is suitable for machine learning tasks. The processed data that were used for the model training were taken after the following preprocessing steps.

##### Step 01—Labeling the Data Set

The videos were of patients with different levels of aphasia. Some of them were very expressive, and some were struggling with pronunciation. As the labeled data set is used for the classification of the patient, the accuracy of labeling had to be very high. Therefore, we have classified the selected videos of each patient into four categories and ten levels by checking each video by the first three authors and a speech therapist using a consensus method. When categorizing a patient into a severity level or category, the presence or absence of features given in Table 1 were checked by the authors and the language therapist.

##### Step 02—Preprocessing of Audio

Then, the audio and the video parts of each video were separated, and the audio parts were split into multiple audio chunks separating the parts where the patient is speaking and the parts where the moderator is speaking. The software tool “Format Factory version 5.5” was used in this splitting, and formats with the highest quality were used in the audio clips. After that, the multiple smaller chunks of each audio where the patient and the moderator were speaking were connected using the Format Factory tool, making a longer audio file containing the speech of the patient and the moderator separately. Then, those combined audio files were separated into 10 s and 20 s chunks for the speech feature extraction process. This was completed because it would give a measure on what should be the ideal size concerning the time for each audio sample that should be inputted to the models. Rather than arbitrarily setting a value, by this approach, it was possible to experiment and see the results for each time split. Then, it is easy to choose a time split which gives highest classification accuracy. The main reason for the 20 s length is that selecting more than a 20 s length per clip results in fewer audio chunks per particular level or category. On the other hand, selecting an audio length shorter than 10 s results in some audio clips containing only talking intervals between few words. As an example, if we select 5 s clips, there are more clips that contain intervals without patients speaking, and for the clips in which the patient speaks, the number of words that the patient speaks is limited to a few words. Table 4 shows the distribution of number of chunks between different aphasia severity levels for 20 s chunks and 10 s chunks.

As evident from Table 4, no audio data are available for level 0 and level 1 in the Aphasia Bank data set, and for level 2, a small number of audio clips is available. For 10 s chunks, each level has more audio clips compared with 20 s chunks. Table 5 shows the distribution of number of chunks between different aphasia categories for 20 s chunks and 10 s chunks.

According to the distribution of the data per category as evident from Table 5, this is not a balanced classification problem, but there are enough data points for each category to complete the classification task. There are more audio data for each category for 10 s than for 20 s. When we split the same audio data into 10 s frames due to the 10 s time frame, more audio data frames can be obtained.

##### Step 03—Feature Engineering Using Speech Feature Extraction

**Figure 2:**
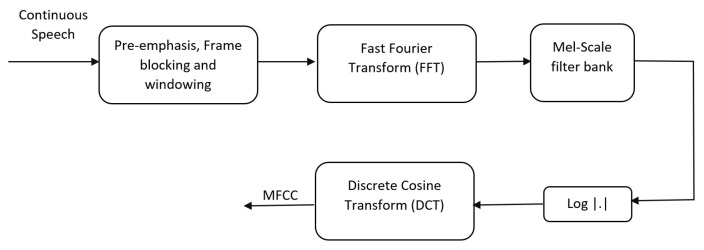
Block diagram for computation of mel frequency cepstral coefficients [35].

The next step of data preprocessing is to extract audio features from the audio clips. Considering the subtleties of the spoken language, it is required to extract the features that contain the most information about the speech. There are a few audio features such as Mel-Spectrogram, Chroma, MFCC, and Zero Crossing Rate (ZCR), which can be extracted using the Librosa library. We experiment on all of these audio features and select the best audio feature based on the classification performance of the machine learning models. Mel-Spectrogram is a representation of the power of audio frequencies in the mel scale to model human hearing perception [88]. Chroma represents the tonal content of a musical audio signal obtained using Short-Time Fourier Transform (STFT) and constant Q transforms [89]. MFFCs are the current industry standard when it comes to audio signal processing. MFCC computation is a replication of the human hearing system intending to artificially implement the ear’s working principle with the assumption that the human ear is a reliable speaker recognizer [35]. MFCC replicates the human hearing system by passing the audio through Fast Fourier Transform (FFT) followed by mel scale filter bank, log scale conversion, and Discrete Cosine Transform (DCT) blocks [35], as shown in Figure 2.

**Figure 3:**
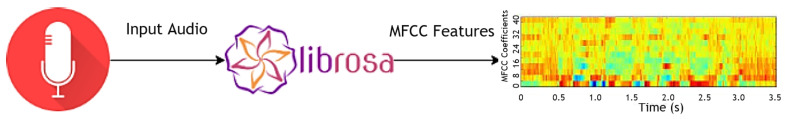
MFCC audio feature extraction.

ZCR is the rate at which the audio signal crosses zero, which represents the noisiness and the spectral characteristics of the signal [90]. We extracted the features for each audio clip at a sampling frequency of 22,050 Hz using a feature extraction layer that was built using the “Librosa” audio processing python library. Audio feature extraction using MFCC is graphically shown in Figure 3.

These extracted audio features were saved in .csv files with the respective labels in order to create the data set required for model training. The data set was then split into training and test sets to train and test the machine learning models. The train to test split ratio is 4:1.

#### 2.6.2. Audio Recording Device

The audio (voice of the person) is gathered with an audio recording device and then converted into the mp3 format. For this task, the recording device should have an audio sensor to convert acoustic energy into electrical energy.

#### 2.6.3. Split the Audio File into 20 S Chunks

The audio file is split into 20 s chunks to be fed into the Librosa library for feature extraction for the real-time system. Here, we select a 20 s time period for data chunks, as 20 s gives a higher classification accuracy over 10 s chunks as evident from the results in Section 3.

#### 2.6.4. Audio Feature Extraction

The mp3 file containing the voice of the subjected person is fed into the Librosa library in python for audio feature extraction. The feature that is used with the model is MFCC, as MFCC gives a higher classification accuracy over other speech feature extractors, as evident from the results in Section 3. The extracted features containing 40 columns of MFCC features are fed into the machine learning classification model.

#### 2.6.5. Machine Learning Classifier

The machine learning model is an audio classification model which is trained to detect the severity of the aphasia level or category using the speech of the patient. The input size will depend on the type of speech feature extractor used. The input size of the machine learning model (output size of the feature extractor) for MFCC, chroma, mel-spectrogram, and ZCR are 40, 259, 40, and 475, respectively. The output has either four classes or ten classes depending on whether the aphasia categories are classified or aphasia levels are classified.

As the data set is labeled, we have to use a supervised machine learning model. Here, we test several supervised machine learning models to be deployed as the classification model. These are

- K-nearest neighbor;
- Decision tree;
- Random forest;
- Deep neural network;

The implementation details of these machine learning approaches are given in the following sections.

##### K-Nearest Neighbor (KNN)

KNN is one of the simplest classification algorithms. It groups the data into coherent clusters and classifies the newly input data based on the similarity of previously trained data [91]. This algorithm exploits the principle that similar things exist in close proximity, so it classifies based on the similarity of the data points according to the computed distance among the data points. Using grid search, we can find that the best metric for the aphasia severity level classification problem is “minkowski” [92], and the number of neighbor sizes should be six. The search range for the neighbor size was from 3 to 12.

##### Decision Tree

A decision tree is a tree-based technique in which any path beginning from the root is described by a data separating sequence until a boolean outcome at the leaf node is achieved [93]. By plotting accuracy score versus the number of leaf nodes, we can find that six leaf nodes give the best classification result for this aphasia severity level classification problem. Therefore, we set the number of leaf nodes as six for this algorithm. We varied the number of leaf nodes from 2 to 10 when finding the optimum value of six.

##### Random Forest

A random forest machine learning model consists of a large number of decision trees where each tree gives out a class prediction, and the class with the highest number of classifications becomes the classification of the model. In a random forest, each node is split using the best among a subset of predictors randomly chosen at that node [94]. In random forest, multiple decision trees are trained and integrated into the result by averaging them. The number of estimator parameters limits the number of decision trees in random forest. Typically, it is kept between 40 and 60.

##### Deep Neural Network (DNN)

**Figure 4:**
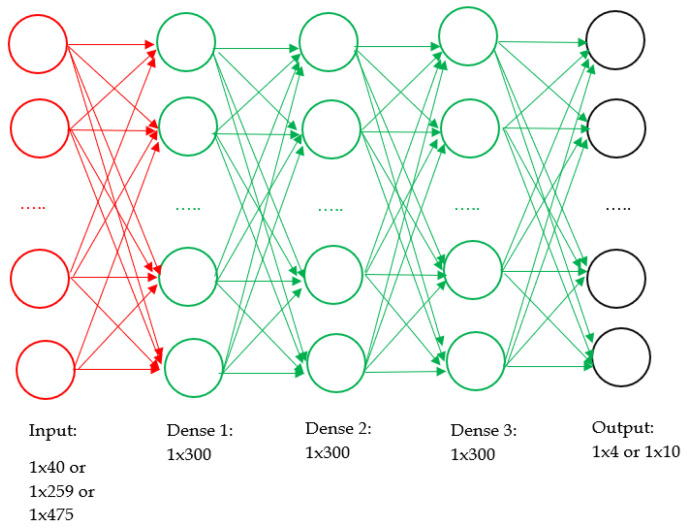
Architecture of the deep neural network used for aphasia classification.

A DNN is an artificial neural network having an input layer, output layer, and one or more hidden layers in between them. Each layer consists of neurons where a weighted summation is carried out to obtain an output. When the DNN is trained, these weights and biases of the neurons are adjusted to minimize a user-defined loss function [95]. DNNs attempt to find a mathematical relationship between the inputs and outputs of the neural network. The architecture of the DNN used in this research is given in Figure 4.

The DNN implemented in this research consists of 3 hidden dense layers. The batch size is typically chosen between one and a few hundred. The batch size of 32 is a good default value according to research conducted in [96]. Therefore, we have set the batch size to 32. There is no way to analytically calculate the number of layers or number of nodes per layer in a neural network for a predictive modeling problem. Other parameters are selected through systematic experimentation and based on the literature. We select rectified Linear Unit (ReLU) as the activation function for inner layers and softmax as the activation function for the output layer, as the output is categorical [97]. The loss function for the neural network is sparse categorical cross-entropy, as the problem is a multi-class classification. We train the DNN at an initial learning rate of 0.001, which decays 4% per epoch for 200 epochs using the Adam optimizer. The initial learning rate of 0.001, learning rate decay rate of 4% per epoch, and the number of neurons of each hidden layer of 300 were found by systematic experimentation (by observing the training curve for 25 epochs). The range of values on which we experimented for the initial learning rate, number of neurons in the hidden layer, and learning rate decay rate are (0.0001, 0.01), (10, 1000), and (0%, 15%), respectively.

##### Performance Evaluation Metrics for Machine Learning Classifiers

For all machine learning classifiers, we evaluate the performance using accuracy, precision, recall, and F1-score. In Equations (1)–(4), TP stands for True Positive, TN stands for True Negative, FN stands for False Negative, and FP stands for False Positive.

Accuracy can be calculated as given in Equation (1). Accuracy is a metric which depicts the correct prediction of outcomes out of all of the predictions. In other words, it measures how often an algorithm classifies a data point correctly. It is useful when all classes are of equal importance.

(1) $$Accuracy\;\left(\%\right)=\frac{TP+TN}{TP+FP+FN+TN}*100$$

However, especially when there is a class imbalanced data set, accuracy alone cannot be used as a metric to evaluate the machine learning model’s classification performance. Other metrics such as precision, recall, and F1-score need to be calculated along with accuracy in such a case in order to obtain an overall idea about the classification performance. Precision can be calculated as given in Equation (2). Precision is a metric that reflects the quality of the positive prediction of the model. In other words, it shows the proportion of the predicted positive classifications which were actually correct out of all of the samples predicted as positives. A high score for the precision indicates that the model’s incorrect prediction for the positive class (false positives) is low, and correct classification for the positive class (true positives) is high.

(2) $$Precision\;\left(\%\right)=\frac{TP}{TP+FP}*100$$

Recall can be calculated as given in Equation (3). Recall is a metric showing the proportion of correctly predicted positives out of all the samples that were actually positive. A high score for the recall indicates that the model’s incorrect prediction for the negative class (false negatives) is low, and correct classification for the positive class (true positives) is high. Therefore, recall is also a metric for the positive prediction performance of a given model such as the precision. In other words, both precision and recall compute the prediction performance of a particular class of interest (positive class). However, precision computes correct positive predictions compared to all positive predictions, while the recall computes positive predictions compared to all actual positive samples. In other words, using precision, we can infer about the false positive rate, and using recall, we can infer about the false negative rate.

(3) $$Recall\;\left(\%\right)=\frac{TP}{TP+FN}*100$$

The F1-score can be calculated as given in Equation (4). The F1-score is calculated using both precision and recall; hence, the effects of both false negatives and false positives are depicted in the F1-score. The F1-score is the harmonic mean of the precision and recall. Harmonic mean discourages largely unequal values and extremely low values: that is, when either the precision or recall are low and close to zero, the F1-score will also be close to zero. Only when precision and recall are both closer to one will the F1-score also be close to one. In aphasia severity level classification, both false positives (classifying a patient from a different class as a given class) and false negatives (classifying a patient from a given class as a different class patient) are undesirable. Hence, the F1-score is expected to be high for this classification problem.

(4) $$F1-score\;\left(\%\right)=2*\frac{Precision*Recall}{Precision+Recall}*100$$

#### 2.6.6. Return the Aphasia Category or Level

The classification model gives the aphasia category or level that the features are mostly correlated with. We test the machine learning models to retrieve either the four aphasia categories or ten aphasia levels. Therefore, we implement a set of models to retrieve categories and another set of models to retrieve the levels for testing purposes.

### 2.7. Development of the Software Application

The classification model is deployed separately from the application for better inference and maintenance. This allows the decoupling of dependencies from the application. The software application follows the Model-View-Controller (MVC) architecture and uses Representational State Transfer (REST) protocol for client–server connectivity. Altogether, a high-level maintenance of the entire system is possible with this clear separation of concerns.

#### 2.7.1. Text to Speech Model Implementation

A Text-to-Speech (TTS) model was developed to facilitate patients to hear the texts displayed on screen. For this implementation, open source implementations for the same were explored. We narrowed the search down to 2 models that are widely being used in the industry at the moment. Those 2 models are Tacotran2 [98] developed by Google and Fast Speech2 [99] developed by Meta AI. After experimenting with these 2 models, it was found that the Tacotran2 model is best suited for the application because of the low latency of the inference and the clean synthetic voice output.

#### 2.7.2. Software Application Architecture

The speech therapy application is designed as a web application that can be accessed by anyone with the Uniform Resource Locator (URL). The application is designed in a way it can keep track of the patient’s level and how they have used the application. If the patient is registered on the platform, they can access the full feature set. If someone needs to identify the severity level of aphasic speech disorder, they can use the aphasia classification model without registering into the web application. This is completed by engaging the user in three exercises as follows.

1. First Exercise: Introduction of themselves around one minute.
2. Second Exercise: Picture description.
3. Third Exercise: Repeating some of the words provided.

**Figure 5:**
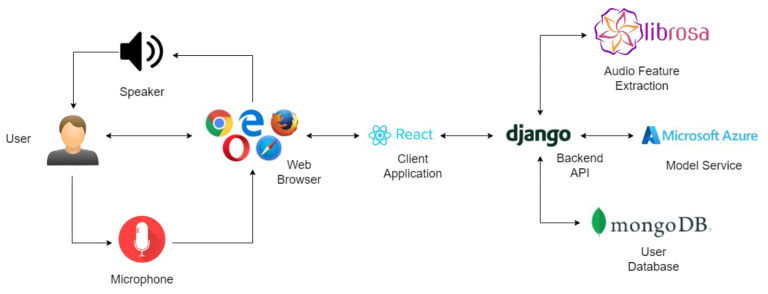
Architecture of the software application for aphasia classification and speech therapy recommendation.

The architectural diagram providing an overview of the web application for automatic aphasia level classification and speech therapy recommendation is shown in Figure 5.

The user interface is delivered to the user via a web browser which can be accessed with any device which is connected to the web. To use the platform effectively, the user should have a microphone and a speaker on the device.

## 3. Results

In this section, we conduct three types of research experiments. First, in Section 3.1, we experiment on different speech feature extraction techniques. Then, in Section 3.2, we experiment on the audio chunk size for both aphasia severity level and category classifications. Finally, in Section 3.3, we experiment on different machine learning techniques for aphasia severity level classification.

### 3.1. Experimenting on Different Audio Feature Extractors for Aphasia Level Classification

In this experiment, we fix the machine learning classifier as a deep neural network for the classification of aphasia audio data into categories and levels, as it will be experimentally proved in Section 3.3 that DNN has the best classification performance. Furthermore, we set the audio chunk size to 10 s due to the availability of a high number of data samples, even though the 10 s chunk size yields a slightly inferior classification performance for level classification than 20 s chunks, as will be evident from Section 3.2. The variable in this experiment is the type of audio feature extracted from the input audio. The following subsections show the aphasia severity level classification performance for different extracted audio features.

#### 3.1.1. Mel-Spectrogram

**Figure 6:**
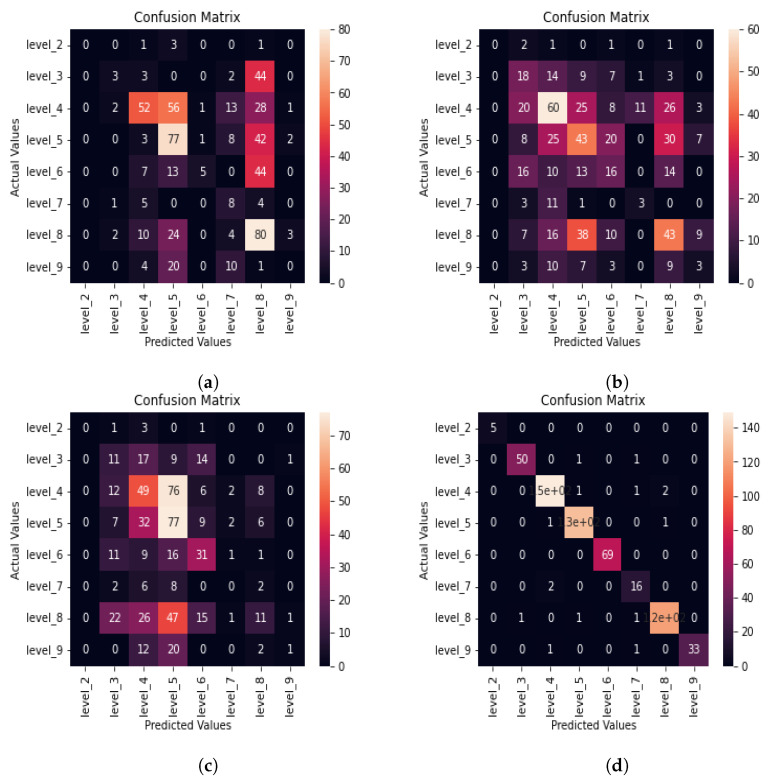
Confusion matrices of deep neural network for aphasia severity level classification using 10 s audio chunks for different speech feature extraction techniques. (**a**) Confusion matrix of DNN whose features have been extracted using mel-spectrogram. (**b**) Confusion matrix of DNN whose features have been extracted using chroma. (**c**) Confusion matrix of DNN with the zero crossing rate audio feature extracted. (**d**) Confusion matrix of DNN whose audio features are extracted using MFCC.

The confusion matrix for the DNN model whose audio features are extracted using mel-spectrogram is shown in Figure 6a.

As evident from the confusion matrix in Figure 6a, many misclassifications can be observed for aphasia level classification when audio features are extracted using mel-spectrogram. We further evaluate the accuracy, precision, recall, and F1-score for the DNN model whose features are extracted using mel-spectrogram, as evident from Table 6.

As evident from the classification report for the DNN model in Table 6, the F1-score for any level does not reach even 0.50 when audio features are extracted using a mel-spectrogram. The precision, recall, and F1-score are zero, and accuracy is also low for level 2 and level 9. Accuracy values are also not close to one for most of the levels. Hence, the mel-spectrogram is less suitable for aphasia level classification tasks due to its lower classification performance.

#### 3.1.2. Chroma

In this section, we investigate the DNN model’s aphasia severity level classification performance when chroma is used as the audio feature. The confusion matrix for aphasia level classification in Figure 6b is for the DNN model when the chroma audio feature is extracted from the aphasic speech.

As evident from the confusion matrix given in Figure 6b, the DNN model is confused, and many misclassifications can be observed. In order to obtain more details about the classification performance, we calculate the accuracy, precision, recall, and F1-score using Equations (1)–(4) to retrieve the classification report of the DNN model when chroma audio features are extracted, as shown in Table 7.

The maximum F1-score is reported by level 4 for the DNN model when the chroma audio feature is extracted, as evident from Table 7. All precision, recall, and F1-score values are less than 0.50 for all levels. This indicates that model’s true positives are low and false predictions are high. Only level 2 and level 7 accuracy are close to one due to the effect of the high number of true negatives. Therefore, the classification performance when chroma is extracted from the aphasic speech is poor.

#### 3.1.3. Zero Crossing Rate

In this section, we investigate the effect of the audio feature zero crossing rate toward the aphasia severity level classification performance. Figure 6c shows the confusion matrix of the DNN when the zero crossing rate audio feature is extracted from the input aphasic speech.

The confusion matrix in Figure 6c also shows a significant number of misclassifications, even though it classifies better compared to the confusion matrix when the chroma audio feature is extracted. We evaluate the classification performance using performance evaluation metrics, as shown in Table 8.

As evident from the classification report in Table 8, the precision, recall, and F1-score are always less than 0.60 for all the levels for the DNN model when the ZCR audio feature is extracted from audio input. Furthermore, all precision, recall, and F1-scores for level 2 and level 7 are zero. This indicates that the model’s true positives are low and false predictions are high. However, level 2, level 7, and level 9 accuracy values are high due to the effect of high true negatives. All other levels’ accuracy values are close to 0.5. Therefore, the aphasia classification performance of DNN when ZCR is used as the audio feature is poor.

#### 3.1.4. Mel Frequency Cepstral Coefficients

In this section, we use 10 s audio chunks for aphasia severity level classification using a DNN whose audio features are extracted using MFCC. Figure 6d shows the confusion matrix of the DNN machine learning model when audio features are extracted using MFCC.

As evident from the confusion matrix given in Figure 6d, the model has correctly predicted most of the test data among the aphasia severity levels except for a few misclassifications. This classification performance is superior to that used when any of the mel-spectrogram, chroma or ZCR are used for audio feature extraction. In order to obtain a better understanding of the classification performance, we use Equations (1)–(4) to calculate the accuracy, precision, recall, and F1-score of the DNN model for aphasia severity level classification whose audio features extracted using MFCC. The calculated metrics are shown in Table 9 in the form of a classification report.

Since precision, recall, and F1-score are greater than or equal to 0.97 for all aphasia levels except for level 7, the DNN model’s predictions consists of a high percentage of true positives and very low percentage of false predictions. Even the level 7 F1-score was 0.84, which is acceptable for level classification. Furthermore, when MFCC is used for audio feature extraction, the DNN model is highly accurate, as evident from accuracy values very close to one for all levels in Table 9. This proves that MFCC is highly suitable to extract features from aphasic speech to classify into the levels defined in this research.

#### 3.1.5. Overall Classification Performance Analysis for Audio Feature Extraction

In this section, we summarize the classification performance when different speech feature extraction techniques are used for aphasic audio feature extraction, as shown in Table 10.

As evident from the classification summary in Table 10, when audio features are extracted using MFCC, the aphasia level classification performance is very high, as all accuracy, precision, recall, and F1-score are close to 1.0. On the other hand, for all other audio feature extractors, the classification performance for aphasic speech is significantly lower than when MFCC is used. Among the other audio features except for MFCC, the zero crossing rate has the least classification performance, while mel-spectrogram has the highest classification performance, as evident from values shown in Table 10.

### 3.2. Experimenting on the Chunk Size for Aphasia Category and Severity Level Classifications

We set the audio feature extractor as MFCC, as it gave the best classification performance, as evident from Section 3.1. Furthermore, we set the machine learning classifier as a deep neural network for the classification of aphasia audio data into categories and levels, as it will be experimentally proved in Section 3.3 that DNN has the best classification performance. The variable in this experiment is the length of the audio clips of aphasia patients, which we vary among 20 s and 10 s.

#### 3.2.1. Audio Chunks Divided into 20 s

##### For aphasia category classification

**Figure 7:**
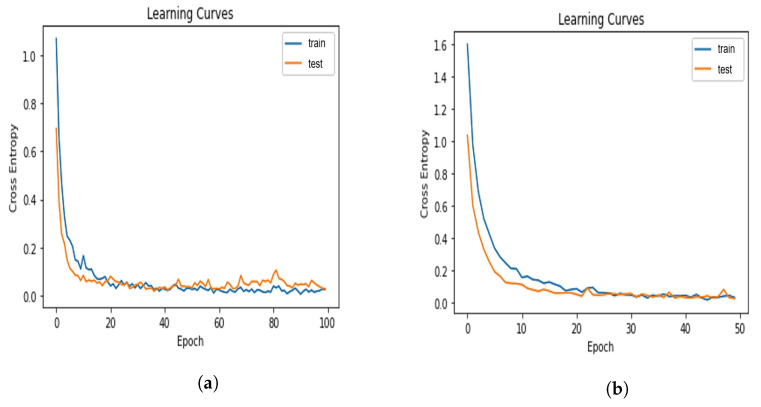

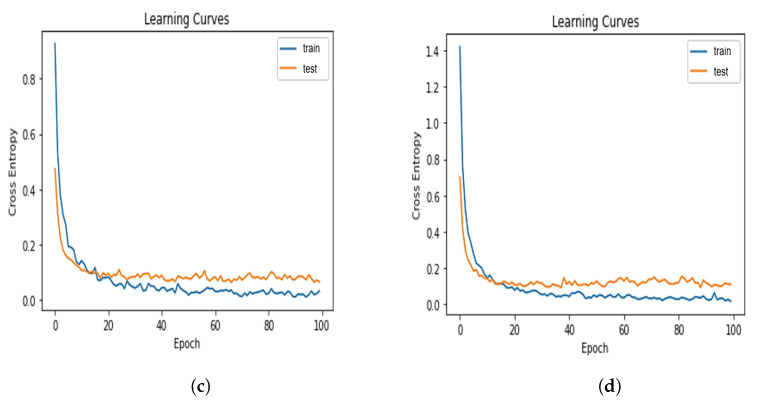
Training and testing curves of DNN model for different aphasia classifications and different input audio lengths whose features have been extracted using MFCC. (**a**) Training and testing curves of DNN model for aphasia category classification using 20 s audio chunks. (**b**) Training and testing curves of DNN model for aphasia severity level classification using 20 s audio chunks. (**c**) Training and testing curves of DNN model for aphasia category classification using 10 s audio chunks. (**d**) Training and testing curves of DNN model for aphasia severity level classification using 10 s audio chunks.

In this section, we set the audio chunk size as 20 s to predict four aphasia categories. Figure 7a shows the training and testing curves of the deep neural network for aphasia category classification, whose audio features have been extracted using MFCC for 20 s chunks.

**Figure 8:**
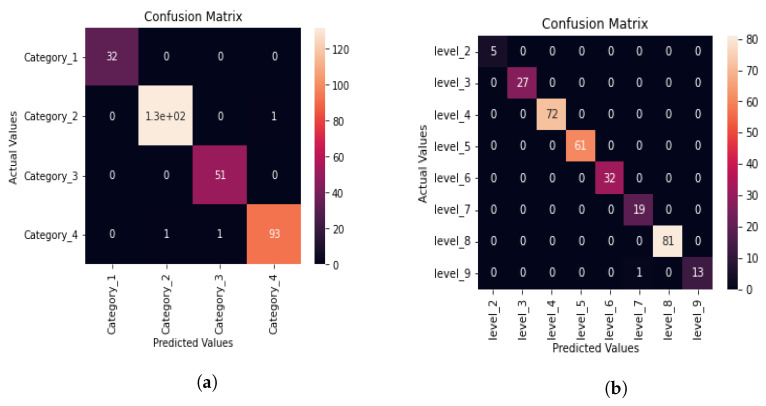

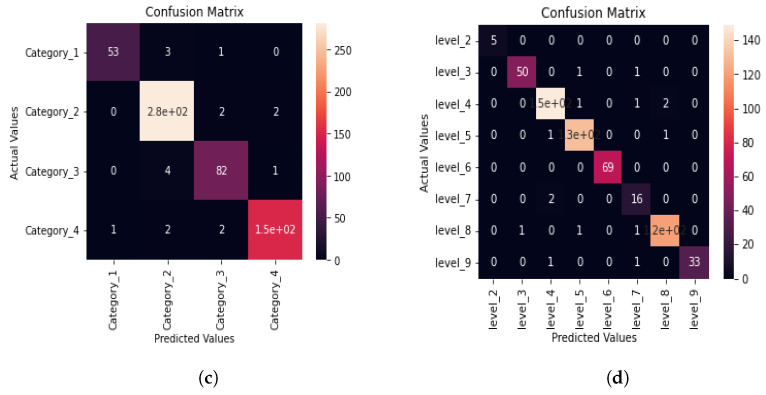
Confusion matrices of DNN machine learning model for different aphasia classifications whose features are extracted using MFCC for audio chunks of different lengths. (**a**) Confusion matrix of DNN for aphasia category classification using 20 s audio chunks. (**b**) Confusion matrix of DNN for aphasia severity level classification using 20 s audio chunks. (**c**) Confusion matrix of DNN for aphasia category classification when 10 s audio chunks are provided as input. (**d**) Confusion matrix of DNN for aphasia severity level classification when 10 s audio chunks are provided as input.

The training curve indicates how well the model is training, and the testing curve indicates how well the model is generalizing. For a good fit, there should be a minimum gap between the training and testing curve. The learning curve for deep neural network which uses 20 s audio chunks for aphasia category classification indicates a good fit with the minimum gap between the training and testing curves, as evident from Figure 7a. Figure 8a depicts the confusion matrix of DNN for aphasia category classification using 20 s audio chunks to extract features using MFCC.

Only three misclassifications can be observed in the confusion matrix given in Figure 8a. Using the confusion matrix obtained in Figure 7a, we can derive a category-wise classification report for the deep learning model having audio features extracted from 20 s chunks, as shown in Table 11. Equations (1)–(4) are used in calculation of the performance evaluation metrics given in Table 11.

As evident from Table 11, all category-wise accuracy, precision, recall, and F1-score are close to 1.0. Therefore, when the length of the audio chunks is 20 s, the DNN model does a very good job at predicting the aphasia categories.

##### For Aphasia Severity Level Classification

In this section, we use 20 s audio chunks for aphasia severity level classification using a DNN. Figure 7b shows the learning curves of the DNN model for aphasia severity level classification when 20 s chunks are used as input and features are extracted using MFCC.

As evident from Figure 7b, the gap between training and testing curves are very low when 20 s chunks are used for aphasia severity level classification which indicates a very good fit of the model for the aphasic data. Figure 8b shows the confusion matrix of DNN model for aphasia severity level classification when 20 s chunks are used and audio features are extracted using MFCC.

The confusion matrix given in Figure 8b is very clean having only 1 misclassification in level 9. This confusion matrix suggests that the aphasia severity level classification performance for 20 s chunks is very good. To confirm the previous statement, we calculate the DNN model performance evaluation metrics for aphasia severity level classification when 20 s speech samples are used, as shown in Table 12 using Equations (1)–(4).

As evident from Table 12, all accuracy, precision, recall, and F1-score values are 1.0 for all levels except level 7 and level 9. Even for level 7 and level 9, the F1-score and accuracy are very close to 1.0. This result indicates that the DNN model has been perfectly fitted for aphasia severity level classification when 20 s audio chunks are used.

#### 3.2.2. Audio Chunks Divided into 10 s

##### For Aphasia Category Classification

In this section, we use 10 s audio chunks for aphasia category classification. Figure 7c shows the training and testing curves for aphasia category classification using 10 s audio chunks whose audio features have been extracted using MFCC.

As evident from the learning curve in Figure 7c, it is a also good fit. However, the gap between the training and testing curve is larger when the audio chunks are 10 s long compared to the gap between the same curves when the audio chunk length is 20s. Therefore, when the input chunk size is 10s, the DNN model’s generalization capability is lower for aphasia category classification. Figure 8c shows the confusion matrix of the DNN model for the aphasia category classification when using 10 s audio chunks as the input whose features have been extracted using MFCC.

In the confusion matrix given in Figure 8c in which 10 s audio chunks are used, more misclassifications can be observed compared with the same when 20 s audio chunks are used. One of the reasons for that can be due to the increment of audio data samples. The confusion matrix alone cannot be used in order to come into a conclusion regarding the performance of the DNN model when the input audio length is changed. For this purpose, we further calculate category-wise the accuracy, precision, recall, and F1-score using Equations (1)–(4) to retrieve the classification report for aphasia categorization when 10 s audio chunks’ features are extracted using MFCC, as shown in Table 13.

Since the F1-score is close to one for all categories, the DNN model’s prediction performance is good as evident from Table 13. However, when comparing the result in Table 13 against the corresponding performance evaluation metrics of the DNN model using 20 s audio chunks as the input given in Table 11, values for the DNN model using 10 s audio chunks are slightly lower than values for the DNN model using 20 s long audio. Therefore, the aphasia category prediction performance is superior when 20 s audio chunks are used.

##### For Aphasia Severity Level Classification

Figure 7d shows the training and testing curves of the DNN model for aphasia severity level classification when features are extracted using MFCC from 10 s audio chunks.

According to Figure 7d, the learning curve is good fit for aphasia severity levels when 10 s audio chunks are used. The gap between the training and testing curve in Figure 7d for aphasia severity level classification using 10 s audio chunks is slightly larger than that for aphasia category classification using 10 s audio chunks shown in Figure 7c. The gap between the training and testing curve in Figure 7d for aphasia severity level classification using 10 s audio chunks is significantly larger than that for aphasia severity level classification using 20 s audio chunks shown in Figure 7b, indicating that by using 20 s speech chunks, the DNN model can generalize well with a perfect fit better than using 10 s audio chunks for aphasia severity level classification.

The DNN confusion matrix for level classification using 10 s chunks is shown in Figure 8d, which is the same as that in Figure 6d in Section 3.1. The classification report is shown in Table 9 in Section 3.1.

#### 3.2.3. Overall Classification Performance Variation with the Length of the Audio Input

Using the preceding results obtained, let us derive an overall classification performance variation with the length of the audio inputs for DNN models for category classification and level classification. Table 14 summarizes the overall classification performance of the DNN model among aphasia severity level and aphasia category classifications for 10 s and 20 s audio chunks.

As evident from the overall classification results in Table 14, for aphasia category classification, the classification performance when using 20 s chunks is superior than the same when using 10 s audio chunks. That is because all the accuracy, precision, recall, and F1-score values for category classification using 20 s are slightly higher than those for aphasia category classification using 10 s chunks. The same can be observed for the aphasia severity level classification also, where the classification performance is better when using 20 s chunks than when using 10 s chunks. Another fact that can be noted from the classification summary in Table 14 is that for chunks of equal length (20 s or 10 s), the overall classification performance of aphasia level classification is better than aphasia category classification, as suggested by the corresponding performance evaluation metrics in Table 14.

### 3.3. Experimenting on Different Machine Learning Classifiers for Aphasia Severity Level Classification

In this section, we fix the audio feature extractor as MFCC, as it was proved in Section 3.1 that feature extraction using MFCC gives the highest classification accuracy. Furthermore, we set the audio chunk size to 10 s due to the prevalence of a high number of data samples, even though a 10 s chunk size yielded slightly inferior classification performance for level classification than 20 s chunks, as it was evident from Section 3.2. The variable in this section is the machine learning classifier block in the aphasia classification model given in Figure 1.

#### 3.3.1. Deep Neural Network (DNN)

**Figure 9:**
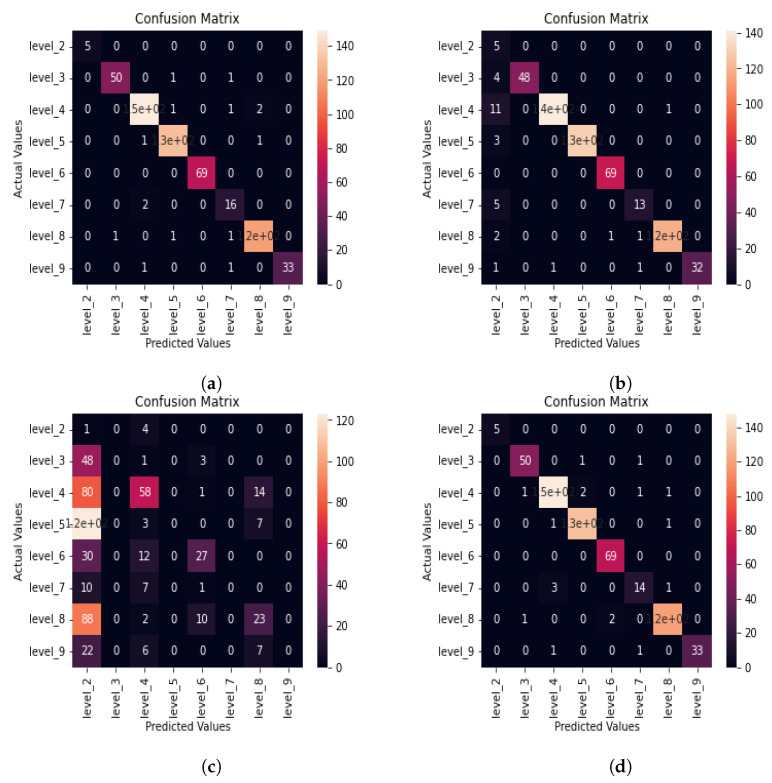
Confusion matrices of different machine learning models for aphasia severity level classification whose features were extracted using MFCC for 10 s long audio input. (**a**) Confusion matrix of DNN. (**b**) Confusion matrix of KNN algorithm. (**c**) Confusion matrix of decision tree algorithm. (**d**) Confusion matrix of random forest classifier.

The confusion matrix of the DNN machine learning model for aphasia severity level classification using 10 s audio chunks whose features were extracted using MFCC is shown in Figure 9a, which is the same as shown in Figure 6d in Section 3.1. The classification report is shown in Table 9 in Section 3.1. A total of 15 misclassifications can be observed for the DNN model, as evident from confusion matrix given in Figure 9a.

#### 3.3.2. K-Nearest Neighbors Algorithm

In this section, we evaluate the classification performance of the K-nearest neighbor algorithm for aphasia severity level classification. The confusion matrix for aphasia severity level classification of the KNN machine learning model using 10 s audio chunks and MFCC for feature extraction is given in Figure 9b.

Figure 9b shows that the KNN machine learning algorithm has 31 misclassifications, which is more than the deep neural network model. To further investigate the classification performance of the KNN algorithm, we calculate the severity level wise of the accuracy, precision, recall, and F1-score using Equations (1)–(4) to obtain the classification report for the KNN machine learning model for aphasia severity level classification, as shown in Table 15.

According to the performance evaluation metrics in Table 15, the precision and F1-score are much lower for level 2 and significantly lower for level 7 compared to the other levels, which are close to 1.0. Furthermore, we can observe that the accuracy, recall, and F1-score for each level given in Table 15 of the KNN machine learning model are lower than the same for DNN given in Table 9. Therefore, we can expect the overall classification performance of DNN to be better than that of the KNN algorithm even though the classification performance of KNN is good.

#### 3.3.3. Decision Tree Algorithm

In this section, we evaluate the performance of the decision tree machine learning algorithm for aphasia severity level classification. Figure 9c shows the confusion matrix of the decision tree machine learning model for aphasia severity level classification using 10 s speech samples whose features have been extracted using MFCC.

As evident from the confusion matrix in Figure 9c, the decision tree machine learning algorithm has much more misclassification than both the deep neural network model and KNN model. To investigate further on the classification performance, we calculate the classification performance evaluation metrics of the decision tree machine learning model for each level using Equations (1)–(4), and summarize as shown in Table 16.

As evident from the classification report in Table 16, recall and F1-score are not close to one for all categories. For some categories such as level 2, level 3, level 7, and level 9, the F1-score is zero, indicating that for those levels, the model has not been able to predict true levels, and false predictions are very high. Most of the accuracy values for levels are also close to 0.5. This result proves that the decision tree algorithm is less suitable for the aphasia severity level classification problem, as its classification performance is much worse than that of the other machine learning models.

#### 3.3.4. Random Forest Classifier

In this section, we investigate the aphasia severity level classification of the random forest machine learning model. Figure 9d shows the confusion matrix for aphasia severity level classification using the random forest classifier having audio features extracted using MFCC for 10 s long audio input.

According to Figure 9d, 18 misclassifications can be observed for the random forest machine learning model. This result shows that the classification performance for the random forest machine learning model is more close to the neural network classification. However, we can still expect a high classification performance for the DNN model, as it had only 15 misclassifications. To further investigate the aphasia severity level classification performance of the random forest machine learning model, we calculate the accuracy, precision, recall, and F1-score using Equations (1)–(4) to obtain the classification report given in Table 17.

As evident from Table 17, the accuracy, recall, precision, and F1-score values are slightly lower than those of the DNN model and higher than those of both the KNN and decision tree machine learning models. The F1-score is close to one in all the levels except for level 7, just like it was for the DNN model. Therefore, for the random forest classifier, we can expect a slightly inferior performance to the DNN model but superior performance to both KNN and random forest classifiers. Using the overall classification performance in the next section, we can further confirm this fact.

#### 3.3.5. Inference on Overall Classification Performance among Different Machine Learning Models

In this section, we calculate the overall accuracy, precision, recall, and F1-score for the aphasia severity level classification of different machine learning algorithms to obtain the summary table given in Table 18.

According to the classification summary among the different machine learning models in Table 18, it is evident that the deep neural network machine learning model has the highest overall accuracy, recall, and F1-score. Only the precision of the DNN model is slightly inferior to that of the KNN model. The classification performance of the random forest classifier is slightly inferior to that of the DNN classifier, as all the metrics of random forest are slightly lower than those of the DNN classifier. The worst classification performance is shown by the decision tree machine learning classifier, as suggested by low values for performance evaluation metrics.

### 3.4. Delivering Speech Therapies Using the Software Application

**Figure 10:**
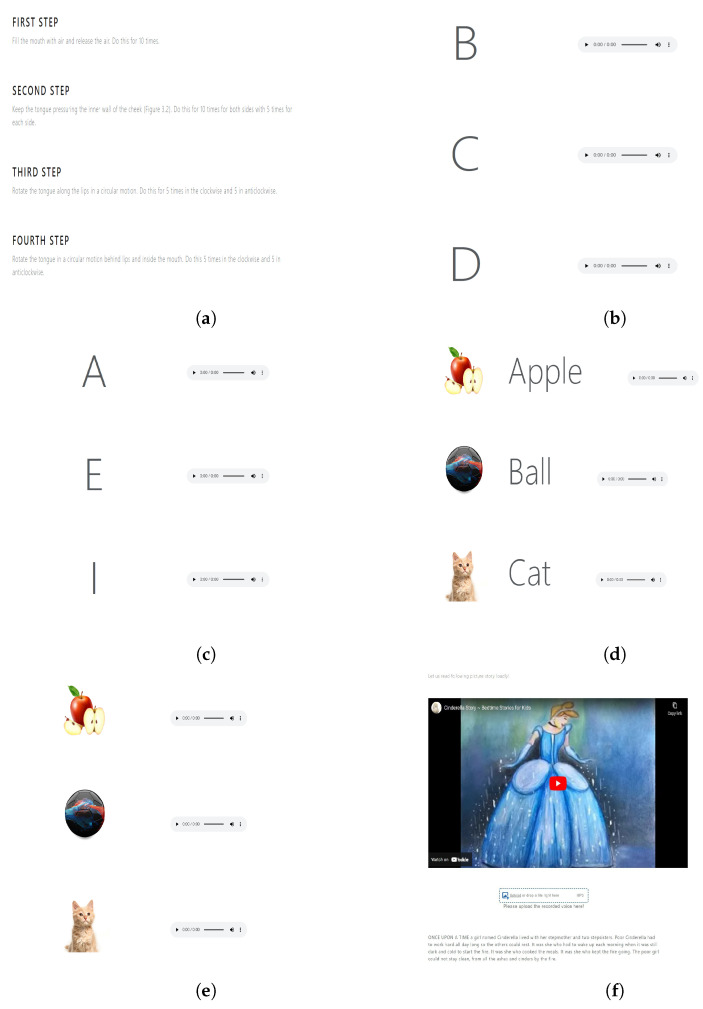
Speech therapy using software application at different aphasia levels. (**a**) Speech therapy at level 0. (**b**) Speech therapy at level 1. (**c**) Speech therapy at level 2. (**d**) Speech therapy at level 3. (**e**) Speech therapy at level 4. (**f**) Speech therapy at level 5.

Figure 10 shows speech therapy exercises shown to the user for different aphasia severity levels. Due to space limitations, we show speech therapies from level 0 to level 5 only.

As evident from Figure 10a, level 0 consists of eight steps that target exercises for the tongue. Level 1 consists of exercises that request the patient to sound the letters as evident from Figure 10b. Level 2 is another step ahead of level 1, which requests the patient to repeat vowel letters, as evident from Figure 10c. In level 3 exercises, the picture of a word is shown along with the word, and then, we encourage the patient to pronounce the word as given in Figure 10d. Level 4 is one step ahead of level 3 where the patient needs to comprehend the picture name when only the picture of an object is shown, as evident from Figure 10e. In level 5, the patient is encouraged to pronounce sentences related to a story, as shown in Figure 10f. The sentences appear as text on screen and first, the sentences are narrated to the patient and then, they are encouraged to pronounce them. The narration occurs either by a pre-recorded audio or by using the text-to-speech converter model implemented in the software. Other levels 6 to 9 are also implemented in a similar approach. When the patient is classified into a particular level by the machine learning model after the initial three tests described in the methodology section, the speech therapies for the identified level will be readily provided to the patient. We use MFCC as the audio feature extractor having 20 s chunks for a deep neural network as the machine learning classifier, as this approach resulted in the highest classification performance, as was evident from previous results section. Thus, by automatic speech assessment of aphasia, speech therapies can be readily provided to the patient using the software application which reduces the workload of speech language therapists by a large margin.

## 4. Discussion

First of all, we will discuss the results of audio feature extraction. It was experimentally proved in Section 3.1 that the use of MFCC for audio feature extraction yields the best aphasia severity level classification performance. The classification performance gaps between MFCC and all other speech feature extraction techniques were large. This result indicates that MFCC is highly suitable for aphasic speech classification into severity levels or aphasia categories. Researchers in [36,37] have used MFCC for feature extraction in an aphasic speech recognition system to investigate the phone error rates of aphasic speech. Another similar study in [100] used MFCC for the feature extraction of Cantonese-speaking aphasia patients to determine syllable error rates. The review paper [101] confirms that most researchers have used MFCC for aphasic feature extraction. However, only a few studies have justified the use of a given audio feature extractor using a performance analysis by comparing it against other audio feature extractors available. In one study [102], three feature spaces—chroma, MFCC, and tonnetz—were tested for impaired speech in specific language impairment where the MFCC gave the highest classification accuracy. In our study for aphasic severity level classification also, MFCC yielded better results than chroma. In aphasic speech assessment, ZCR has been used to extract features as evident from the study conducted in [103]. Mel-spectrograms have also been used to detect features in impaired speech, as evident from the research completed in [104]. In this study, we proved that MFCC outperforms the mel-spectrogram, chroma, and zero crossing rate audio feature extraction techniques. However, for aphasic Arabic-speaking patients, the use of mel-spectrograms has resulted in a slightly higher accuracy than MFCC for a convolutional neural network which performs aphasic speech assessment [105]. Our results contradict this study, as mel-spectrograms received the second highest classification performance among the audio feature extractors compared, being second to MFCC by a large performance gap for our data set, which consists of English-speaking aphasia patients. However, our results cannot be directly compared with research conducted in [105], as we have not used a CNN for aphasic speech assessment, and the language used by us is English, and researchers in [105] have used Arabic.

Many aphasia speech recognition systems deal in speech assessment in syllable or phonum durations [36,37]. According to the best of our knowledge, nobody has conducted research to investigate the optimal input audio chunk length for aphasia severity level or aphasia category detection. In Section 3.2, we experimentally proved that in the neural network approach using MFCC, for both aphasia category classification and aphasia severity level classification, 20 s long speech data sets show slightly better classification results than 10 s speech data. Furthermore, both data sets gave very good classification results, and the performance gap between the two sets is very low. Even though the number of the data samples is twice as high in 10 s than in 20 s, the obtained result shows that 20 s data chunks outperform the corresponding aphasia category and aphasia severity level classification results for 10 s, even for the aphasia severity levels or aphasia categories with a small number of data samples. Furthermore, the number of misclassifications is less in aphasia severity level classification compared with aphasia category classification. Using this result, we can infer 20 s as the ideal audio input length for aphasic category or aphasia severity level classification. That can be due to the fact that 20 s of audio can contain multiple sentences spoken by the patient with aphasia. Within 10 s, the patient can speak only one or two sentences. Therefore, by using the features extracted using 20 s audio, the machine learning model can generalize better than 10 s audio inputs.

Some recent studies have compared different machine learning techniques for aphasia category classification. Nobody has conducted a comparison for aphasia severity level classification, as severity levels are introduced in this research. In study [106], the authors have evaluated the performance of ML techniques for aphasia category classification where the decision tree algorithm has resulted in the highest classification accuracy followed by KNN. However, according to our results in Section 3.3, decision tree has the worst classification accuracy, which contradicts the previous study. In the decision tree classification algorithm, the results were very poor in our study, where the number of misclassifications was higher than the number of correct classifications. A previous study [106] does not evaluate the use of deep neural network. A recent study evaluating the performance of ML models for aphasia assessment reports that the random forest classifier has a better classification performance than the KNN algorithm [107]. Our study confirms the finding of the previous study, as the random forest classifier had an overall accuracy of 0.9849 and the KNN classifier had an accuracy of 0.9833 for aphasia severity level classification using 10 s speech chunks whose audio features have been extracted using MFCC. In our study, KNN gives good classification results, but there are more misclassifications in level 2 and level 7. However, the deep learning technique used in [107] cannot be compared with our work, as it has a convolutional neural network representing audio in time and frequency domains, which is different to the DNN used in our work. The random forest classification results are more close to the neural network, but in level 7, which has a few data samples, the neural network classification results are a little bit better than those of random forest. This proves that for multi-class classification using audio data, an artificial neural network gives better results than conventional machine learning algorithms. The study in [108] attempts to classify aphasia speech into binary classes where the classification performances of support vector machines and naive Bayes have been examined. This work cannot be directly compared with ours, as it involves only two aphasia classes, and we do not investigate the performance of support vector machines and naive Bayes machine learning models. According to the study in [109], the deep neural network model achieves the highest classification accuracy of 80% for classifying primary progressive aphasia into three classes. In this research, which we classify into multiple aphasia severity levels (eight levels due to lack of data in level 0 and level 1), the DNN machine learning classifier yielded the highest overall accuracy of 0.9999, whose input features were extracted using MFCC for 20 s long audio chunks.

As it was reviewed in the literature, digital solutions such as camlender [12], vithea [13], EVA park [14], Everyday Life Activities (ELA) [16], and Web-ORLA [17] exist for aphasia patients to participate in conversation, engage in speech practice sessions, engage in social interactions, etc. using different techniques. However, none of these digital solutions identify the aphasia severity level and recommend speech therapies accordingly. The identification of severity level is very important in order to provide specific treatment for the patient. Furthermore, none of the previously discussed digital solutions provide speech therapies for global aphasia patients. Almost all applications in the literature are designed for aphasia level 3 and onwards. The software solution developed by us is capable of diagnosing the aphasia severity level and providing the specific speech therapies applicable to that level, which will increase the effectiveness of the therapy.

Our findings will be very valuable to the researchers who investigate the assessment of aphasic speech by selecting the audio feature extraction technique, the length of the input aphasic speech for assessment, and the machine learning classifier. The web application will be valuable to the English-speaking patients all around the world to engage in specific speech therapies after automatic assessment of their aphasic speech, which will drastically reduce the workload of the speech language therapists and reduce costs for training. By identifying the correct aphasia severity level from the patient’s voice, it opens doors for the aphasia patients to engage in specific speech therapies at home using personalized home training sessions, which will increase the therapy engagement time and improve the rehabilitation process of the patient.

## 5. Conclusions

In this research, we identified ten aphasia severity levels which map to specific speech therapies. The identification of aphasia speech level is essential for a patient’s speech recovery using speech therapies. This research investigated the performance of several speech feature extraction techniques, length of input audio, machine learning model toward classification performance of aphasia severity levels and aphasia categories. As per the results, audio feature extraction using MFCC outperformed other audio preprocessing techniques such as mel-spectrogram, chroma, and ZCR by a large margin. It can be concluded that using 20 s aphasic speech samples results in a higher classification performance than using 10 s samples, even though the performance gap is very narrow. Finally, the deep neural network’s classification performance is slightly better than that of the random forest and KNN classifiers, and it is significantly better than that of the decision tree algorithm. Therefore, it can be concluded that by audio feature extraction using MFCC for 20 s long speech samples using a deep neural network as the machine learning classifier, aphasia severity levels can be automatically identified very accurately, as it resulted in overall accuracy, precision, recall, and F1-score values of 99%. Therefore, the preceding approach is much more capable of adapting to the subtleties of aphasic speech. By using the proposed approach in a software application, the patients with aphasia can quickly classify themselves into the respective aphasia level, so that the patient’s recovery can be started within a short amount of time without any involvement of a speech language therapist.

## 6. Limitations and Future Work

We could not find patients for the aphasia level 0 and level 1 in the Aphasia Bank database possibly because they are the most severe levels of aphasia, and patients are rare. Therefore, the classification results contain classification into eight levels only. However, still for global aphasia, we have categorized into two sub-levels, level 2 and level 3, which none of the other researchers have done. We have considered only English-speaking aphasia patients. However, it can be readily extended to other languages as well, and we can expect similar results for those languages because the levels defined by us are not language specific. The speech defect levels exist for any aphasia patient speaking any language. The study does not involve live aphasia patients. As future work, the implemented software can be tested using live aphasia patients to evaluate its effectiveness.

## Figures and Tables

**Table 1:** Mapping of aphasic speech severity level with speech features.

Feature\Level	0	1	2	3	4	5	6	7	8	9	10
SF	✓	✓	✓	✓	✓	✓	✓	✓	✓	✓	✓
LM	✓	✓	✓	✓	✓	✓	✓	✓	✓	✓	✓
TM	✓	✓	✓	✓	✓	✓	✓	✓	✓	✓	✓
AS	✗	✓	✓	✓	✓	✓	✓	✓	✓	✓	✓
RSTS	✗	✗	✓	✓	✓	✓	✓	✓	✓	✓	✓
RVS	✗	✗	✗	✓	✓	✓	✓	✓	✓	✓	✓
RWOCS	✗	✗	✗	✗	✓	✓	✓	✓	✓	✓	✓
PCWOS	✗	✗	✗	✗	✗	✓	✓	✓	✓	✓	✓
RGSWE	✗	✗	✗	✗	✗	✗	✓	✓	✓	✓	✓
RSSCWE	✗	✗	✗	✗	✗	✗	✗	✓	✓	✓	✓
RSWAQ	✗	✗	✗	✗	✗	✗	✗	✗	✓	✓	✓
RCWE	✗	✗	✗	✗	✗	✗	✗	✗	✗	✓	✓
RCAF	✗	✗	✗	✗	✗	✗	✗	✗	✗	✗	✓

**Table 2:** Grouping aphasia severity levels into categories and aphasia types.

Category	Levels	Aphasia Type
Category 1	0, 1, 2, 3	Global Aphasia
Category 2	4, 5	Broca’s Aphasia
Category 3	6, 7	Wernicke’s Aphasia
Category 4	8, 9	Anomic Aphasia

**Table 3:** Mapping of aphasia severity levels to speech therapies.

Level	Speech Therapy
Level 0	Fill the mouth with air and release the air, keep the tongue pressuring the inner wall of the cheek [63]
Level 1	Produce sounds from the throat which does not involve tongue (AA, HAA) [64]
Level 2	Encourage the patient to produce sounds that involve both the tongue and the lips (WAA, MAA, say cheese) [63]
Level 3	Show a picture of a letter (F, H, Q) or a picture of an object containing the name of the object below the object and ask the patient to pronounce the letter [65]
Level 4	Sound the name of the objects when a picture of the object is shown without having the name of the object shown below [66]
Level 5	Pronounce the sentences in the stories clear and aloud to the patient. Give the patient the chance to pronounce the same sentences [67]
Level 6	Give the patient a chance to sing the song with the aid of the lyrics [68]
Level 7	Ask simple questions from the patient which can be answered with a single word [69]
Level 8	Ask something from the patient which the patient requires to form sentences to answer [70]
Level 9	Encourage the patient to have a conversation [71]
Level 10	No treatment is required at this stage

**Table 4:** Distribution of data among different aphasia severity levels for 10 s chunks and 20 s chunks.

Category	No. of 10 s Chunks	No. of 20 s Chunks
Level 0	0	0
Level 1	0	0
Level 2	40	25
Level 3	270	130
Level 4	695	340
Level 5	700	350
Level 6	380	190
Level 7	140	80
Level 8	570	370
Level 9	145	70

**Table 5:** Distribution of data among different aphasia categories for 10 s chunks and 20 s chunks.

Category	No. of 10 s Chunks	No. of 20 s Chunks
Category 1	310	155
Category 2	1395	690
Category 3	520	270
Category 4	715	440

**Table 6:** Classification report of DNN model for aphasia level classification using 10 s audio chunks whose features are extracted using mel-spectrogram.

Aphasia Level	Accuracy	Precision	Recall	F1-Score
Level 2	0.40	0.00	0.00	0.00
Level 3	0.81	0.38	0.06	0.10
Level 4	0.63	0.61	0.34	0.44
Level 5	0.57	0.40	0.58	0.47
Level 6	0.77	0.71	0.07	0.13
Level 7	0.83	0.18	0.44	0.25
Level 8	0.52	0.33	0.65	0.44
Level 9	0.37	0.00	0.00	0.00

**Table 7:** Classification report of DNN model for aphasia severity level classification using 10 s audio chunks whose features are extracted using chroma.

Aphasia Level	Accuracy	Precision	Recall	F1-Score
Level 2	0.97	0.00	0.00	0.00
Level 3	0.67	0.23	0.35	0.28
Level 4	0.51	0.41	0.39	0.40
Level 5	0.50	0.32	0.32	0.32
Level 6	0.65	0.25	0.23	0.24
Level 7	0.88	0.20	0.17	0.18
Level 8	0.53	0.34	0.35	0.35
Level 9	0.79	0.14	0.09	0.11

**Table 8:** Classification report of the DNN model for aphasia level classification using 10 s audio chunks whose features are extracted using the zero crossing rate.

Aphasia Level	Accuracy	Precision	Recall	F1-Score
Level 2	0.97	0.00	0.00	0.00
Level 3	0.65	0.17	0.21	0.19
Level 4	0.46	0.32	0.32	0.32
Level 5	0.43	0.30	0.58	0.40
Level 6	0.69	0.41	0.45	0.43
Level 7	0.88	0.00	0.00	0.00
Level 8	0.58	0.37	0.09	0.14
Level 9	0.84	0.33	0.03	0.05

**Table 9:** Classification report of DNN model for aphasia severity level classification using 10 s audio chunks whose audio features are extracted using MFCC.

Aphasia Level	Accuracy	Precision	Recall	F1-Score
Level 2	1.00	1.00	1.00	1.00
Level 3	0.99	0.98	0.96	0.97
Level 4	0.97	0.97	0.97	0.97
Level 5	0.99	0.98	0.98	0.98
Level 6	1.00	1.00	1.00	1.00
Level 7	0.99	0.80	0.89	0.84
Level 8	0.99	0.98	0.98	0.98
Level 9	0.99	1.00	0.94	0.97

**Table 10:** Overall classification report of DNN machine learning model for aphasia level classification using different speech feature extraction techniques for 10 s audio chunks.

Audio Feature Extractor	Accuracy	Precision	Recall	F1-Score
Mel-spectrogram	0.6048	0.4775	0.4155	0.3967
Chroma	0.5423	0.3450	0.3404	0.3428
Zero crossing rate	0.5169	0.3161	0.3350	0.2963
Mel frequency cepstral coefficients	**0.9851**	**0.9749**	**0.9736**	**0.9741**

**Table 11:** Category-wise classification report of DNN model using 20 s audio chunks.

Aphasia Category	Accuracy	Precision	Recall	F1-Score
Category 1	1.00	1.00	1.00	1.00
Category 2	0.99	0.99	0.99	0.99
Category 3	0.99	0.98	1.00	0.99
Category 4	0.99	0.99	0.98	0.98

**Table 12:** Classification report of DNN model for aphasia severity level classification using 20 s audio chunks.

Aphasia Level	Accuracy	Precision	Recall	F1-Score
Level 2	1.00	1.00	1.00	1.00
Level 3	1.00	1.00	1.00	1.00
Level 4	1.00	1.00	1.00	1.00
Level 5	1.00	1.00	1.00	1.00
Level 6	1.00	1.00	1.00	1.00
Level 7	0.98	0.95	1.00	0.97
Level 8	1.00	1.00	1.00	1.00
Level 9	0.98	1.00	0.93	0.96

**Table 13:** Aphasia category-wise classification performance evaluation metrics of DNN model when 10 s audio chunks are used.

Aphasia Category	Accuracy	Precision	Recall	F1-Score
Category 1	0.99	0.98	0.93	0.95
Category 2	0.98	0.97	0.99	0.98
Category 3	0.98	0.94	0.94	0.94
Category 4	0.99	0.98	0.97	0.97

**Table 14:** Overall classification report of DNN models for aphasia severity level and aphasia category classifications using different lengths of audio chunks.

Classification Type and Chunk Size	Accuracy	Precision	Recall	F1-Score
Level classification using 10 s chunks	0.9851	0.9749	0.9736	0.9741
Level classification using 20 s chunks	**0.9999**	**0.9992**	**0.9979**	**0.9984**
Category classification using 10 s chunks	0.9831	0.9692	0.9714	0.9684
Category classification using 20 s chunks	**0.9930**	**0.9894**	**0.9896**	**0.9880**

**Table 15:** Aphasia severity level classification report of KNN machine learning model using 10 s audio chunks and MFCC feature extractor.

Aphasia Level	Accuracy	Precision	Recall	F1-Score
Level 2	0.98	0.16	1.00	0.28
Level 3	0.99	1.00	0.92	0.96
Level 4	0.97	0.99	0.92	0.96
Level 5	0.99	1.00	0.98	0.99
Level 6	0.99	0.99	1.00	0.99
Level 7	0.99	0.87	0.72	0.79
Level 8	0.99	0.99	0.97	0.98
Level 9	0.99	1.00	0.91	0.96

**Table 16:** Aphasia severity level classification report of decision tree machine learning model using 10 s audio chunks having features extracted using MFCC.

Aphasia Level	Accuracy	Precision	Recall	F1-Score
Level 2	0.18	0.00	0.20	0.00
Level 3	0.68	0.00	0.00	0.00
Level 4	0.46	0.62	0.38	0.47
Level 5	0.35	0.00	0.00	0.00
Level 6	0.66	0.64	0.39	0.49
Level 7	0.86	0.00	0.00	0.00
Level 8	0.46	0.45	0.19	0.26
Level 9	0.76	0.00	0.00	0.00

**Table 17:** Aphasia severity level classification report of random forest machine learning model using 10 s audio chunks whose features are extracted using MFCC.

Aphasia Level	Accuracy	Precision	Recall	F1-Score
Level 2	1.00	1.00	1.00	1.00
Level 3	0.97	0.96	0.96	0.96
Level 4	0.97	0.97	0.97	0.97
Level 5	0.99	0.98	0.98	0.98
Level 6	0.99	0.97	1.00	0.99
Level 7	0.99	0.82	0.78	0.80
Level 8	0.99	0.98	0.98	0.98
Level 9	0.99	1.00	0.94	0.97

**Table 18:** Overall classification report of different machine learning models for aphasia severity level classification using audio features extracted using MFCC for 10 s audio chunks.

Machine Learning Model	Accuracy	Precision	Recall	F1-Score
Deep Neural Network	**0.9851**	0.9749	**0.9736**	**0.9741**
K-Nearest Neighbor	0.9833	**0.9877**	0.9478	0.9680
Decision Tree	0.4644	0.3767	0.2171	0.2721
Random Forest	0.9849	0.9725	0.9719	0.9725

## Data Availability

Not applicable.
